# The horizontal shear fracture of the pelvis

**DOI:** 10.1007/s00068-021-01764-3

**Published:** 2021-08-02

**Authors:** Brenton P. Johns, Zsolt J. Balogh

**Affiliations:** 1 Department of Traumatology, John Hunter Hospital, Newcastle, NSW Australia; 2 Discipline of Surgery, School of Medicine and Public Health, University of Newcastle, Newcastle, NSW Australia

**Keywords:** Pelvic fracture, Acetabulum, Trauma, Injury, Horizontal shear

## Abstract

**Purpose:**

Various classification systems describe fractures of the acetabulum and pelvis separately. Horizontal shear fractures involve the pelvic ring and both acetabula and have not been previously described. The aim of this study is to describe the horizontal shear fracture of the pelvis.

**Methods:**

At a level 1 trauma centre over 10 years from December 2008 to December 2018, 1242 patients had pelvic and acetabular fractures. Six patients had horizontal shear fractures, comprising 0.5% of all pelvic and acetabular fractures. Demographic, clinical and radiological data was collected. Clinical outcomes were pain and mobility level, sciatic nerve symptoms, further acetabular or pelvic surgery, or total hip arthroplasty. Radiological outcomes included fracture displacement, implant migration, femoral head osteonecrosis, and post-traumatic arthritis. Outcomes were assessed at a minimum 12 month follow-up.

**Results:**

The median patient age was 35 years. Five of six shear fractures were due to motorcycle crashes. No mortalities occurred. At follow-up, three patients reported pain, two patients had difficulty mobilising associated with traumatic sciatic nerve injury, and one patient underwent total hip arthroplasty for femoral head osteonecrosis. No fracture displacement or implant migration occurred. The Matta arthritis grade was excellent or good in all except one hip. Median follow-up time was 1.8 (range 1.1–7.8) years.

**Conclusion:**

The horizontal shear fracture of the pelvis is a high-energy injury characterised by separation of the anterior and posterior pelvic ring through the acetabula. Good outcomes can be achieved with open reduction and internal fixation of displaced fractures.

## Introduction

Pelvic ring and acetabular fractures are often considered separately in current classification systems. For acetabular fractures, Letournel described the classic elementary and associated fracture types [1], whilst for pelvic ring injuries, the Tile stability-related classification [2] or Young and Burgess [3] mechanism-related classification is commonly used. Both pelvic and acetabular fractures are also included in comprehensive fracture classifications such as the Arbeitsgemeinschaft für Osteosynthesefragen (AO) Foundation and Orthopaedic Trauma Association (OTA) systems [4]. In patients with acetabular fractures, 1% are bilateral [5]. Specifically, bilateral transverse type acetabular fractures have only been described in two case reports previously [6, 7]. We have observed a unique type of fracture, the horizontal shear pattern which has not yet been reported. These are bilateral transverse acetabular fractures separating the anterior and posterior pelvic ring through the acetabula. This study’s objective is to describe the horizontal shear fracture, associated clinical features, subsequent management, and outcomes associated with this pattern.

## Materials and methods

All patients with pelvic and acetabular fractures from a level 1 trauma centre’s prospective pelvic and acetabular fracture database from December 2008 to December 2018 were retrospectively reviewed to identify the patients of interest. The database contains patient medical record numbers, age, sex, and whether a pelvic or acetabular fracture was sustained. Radiological images for all patients were reviewed using our institutions radiology imaging system. This included radiographs, computerised axial tomography (CT) scans, magnetic resonance imaging scans, and bone scans. The key inclusion criterion was any patient with the horizontal shear fracture pattern. This was defined by bilateral transverse type acetabular fractures (OTA classification fracture type 62B1) with separation of the anterior and posterior pelvic ring through these acetabulum fractures. The transverse pattern was defined by Letournel’s classification [1]. Patients with bilateral transverse acetabular fractures who additionally sustained associated posterior wall fracture, comminuted fracture pattern, or pelvic ring fracture were included. All other pelvic and acetabular fracture types were excluded.

Detailed demographic data, aetiology, clinical features, investigation results, and management were collected retrospectively for each included patient via our institution's digital clinical information system. The injury severity score was recorded and patients were classified as polytrauma victims by the Berlin definition [8]. Patients were investigated with pre-operative radiographs and CT scanning of the pelvis with 3-dimensional reconstructions. The images of patients with bilateral transverse acetabular fractures were reviewed in detail to describe the fracture pattern. Fractures were classified into infratectal (62B1.1), juxtatectal (62B1.2), or transtectal (62B1.3) types according to Letournel’s classification [1]. Clinical and radiological outcomes were recorded at the patient’s most recent follow-up after history, examination, and pelvis radiographs were performed. Minimum follow-up required was 12 months.

Clinical outcomes included pain and mobility level adapted from Matta [5], sciatic nerve symptoms, further acetabular or pelvic surgery, other related surgeries, or total hip arthroplasty. These outcomes were patient reported. Sciatic nerve symptoms are specifically related to weak ankle dorsiflexion or “foot-drop”. Related surgeries were any pertaining to the bilateral acetabular fractures. Radiological outcomes measured on follow-up radiographs included post-traumatic hip arthritis, femoral head osteonecrosis, fracture displacement, and implant migration. Post-traumatic hip arthritis was assessed on plain X-ray as described by Matta’s classification [5]. Femoral head osteonecrosis was defined on radiographs using the updated Ficat classification [9]. Heterotopic ossification was assessed and graded according to the Brooker classification [10].

Institutional ethics waiver was obtained prior to completing this study (Reference Number: AU201908-07). Patients were contacted and consent was obtained to utilise their images for the illustrative purposes of the study. Detailed statistical analysis was not required for this study. One patient was lost to local long-term follow-up as they were reviewed at a different hospital beyond 2 months post-operatively and thus were not included in the outcomes reported at follow-up.

## Results

### Demographics

**Fig. 1:**
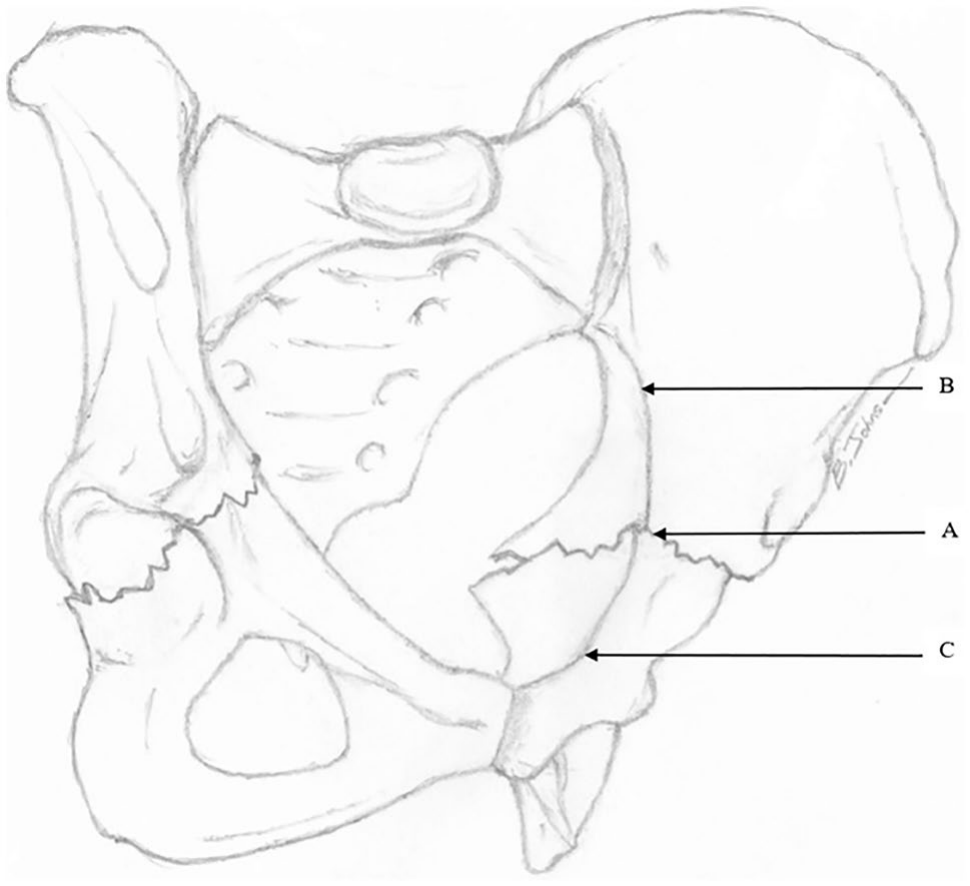
The horizontal shear fracture pattern demonstrated on oblique view of the pelvis. As the acetabulum lies at the junction of the anterior and posterior ring, bilateral transverse acetabular fractures (**a**), separate the posterior ring (**b**), and anterior ring (**c**). Note the horizontal displacement of the anterior ring relative to the posterior ring

During the 10-year period, 1242 patients had pelvic or acetabular fractures. Acetabular fractures affected 283 patients. Six patients had horizontal shear pelvic fractures, representing an incidence of 0.5% of all pelvic and acetabular fractures (see Fig. 1). All six were included in this study. Five patients were young males in motorcycle crashes. This involved going over the handlebars and in one case T-boning a car. Estimated crash speeds ranged from 45 to 80 kms per hour. One young female was crushed by a horse. The median age was 35.5 (range 17–49) years. See Table 1 for demographic data.

**Table 1:** Demographics, associated injuries, and clinical features

	Number of patients (*n*)
*Demographics*	
Age (years)^a^	35.5 (range 17–49)
Male: female (*n*)	5:1
Australian ethnicity (*n*)	6
Smoker (*n*)	1
Employed (*n*)	5
Mechanism (*n*)	
Motorcycle crash	5
Crushed by horse	1
Previous hip or acetabular surgery (*n*)	0
Injury Severity Score^a^	8.5 (range 4–34)
Polytrauma^b^ (*n*)	2
*Associated injuries and clinical features*	
Sustained other injuries (*n*)	6
Sciatic nerve injury (*n*)	3 (bilateral in 1)
Posterior pelvic ring injury (*n*)	4
Hip dislocation (*n*)	1
Femoral shaft fracture (*n*)	1
Lumbar vertebral fractures (*n*)	2
Thoracic fractures (*n*)	2 (1 rib, 1 sternum)
Upper limb fractures (*n*)	3 (1 humerus, 1 scapula, 1 scaphoid)
Bladder injury (*n*)	0
Intracranial haemorrhage (*n*)	1
Blood pressure on admission (mmHg)^a^	126/72
Heart rate on admission (beats per min)^a^	120
Intensive care unit admission (*n*)	2
Massive transfusion protocol (*n*)	2
Pelvic angioembolisation (*n*)	1
*AIS body region*	*Number of injuries with score* ≥ *2*
1—Head and neck (*n*)	1
2—Face (*n*)	2
3—Thorax (*n*)	6
4—Abdomen (*n*)	1
5—Extremity *(n)*	20
6—External (*n*)	0

*AIS* Abbreviated Injury Scale. Note pelvic and acetabular fractures included in Body Region 5: Extremity

^a^Value given as median

^b^Based on International consensus ‘Berlin definition’

### Clinical presentation and associated injuries

On presentation, patients could not weightbear. Four patients were not intubated and had bilateral pelvic pain and tenderness, and were haemodynamically stable. The other two patients were intubated and required massive transfusion protocol (MTP) activation. One had an MTP after suffering a haemothorax and a proximal femoral shaft fracture managed with intramedullary nailing while another required angioembolisation for internal iliac artery bleeding. Three patients sustained a sciatic nerve injury including one neuropraxia and two with loss of ankle dorsiflexion or “foot-drop”. Posterior pelvic ring injuries included unilateral sacral alar fractures in three patients and a unilateral anterior sacroiliac joint (SIJ) injury in one patient. No posterior pelvic ring injury required definitive fixation as sacral alar fractures were undisplaced, the SIJ injury was anterior only, and patients were kept non-weightbearing for 6 weeks. All upper limb fractures were managed non-operatively. No bladder injuries were found on examination, following urinary catheterisation or on pelvis CT scan. The median hospital length of stay was 20.5 days (range 8–32 days).

### Radiological investigations

**Fig. 2:**
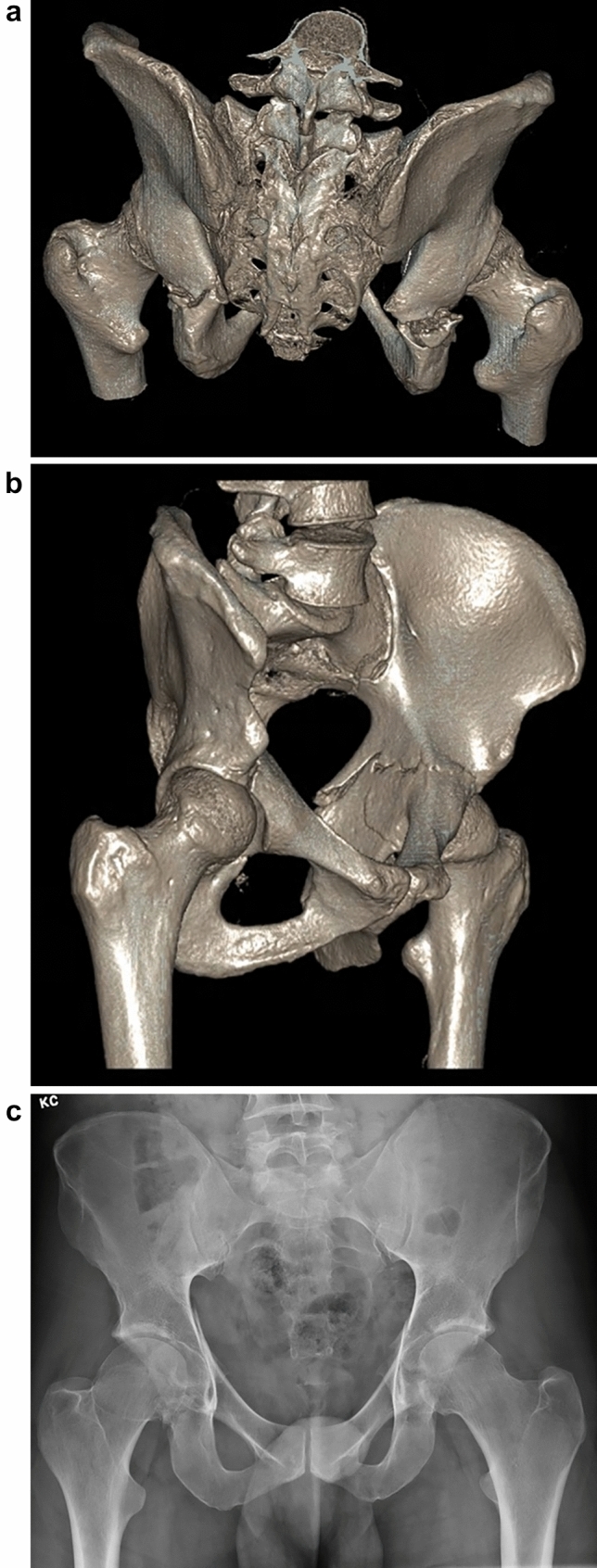
3D surface-rendered CT reconstruction images; posterosuperior view (**a**), right oblique view (**b**), demonstrating posterior displacement of the anterior ring and antero-posterior pelvis radiograph (**c**) demonstrating bilateral transverse acetabular fractures

**Fig. 3:**
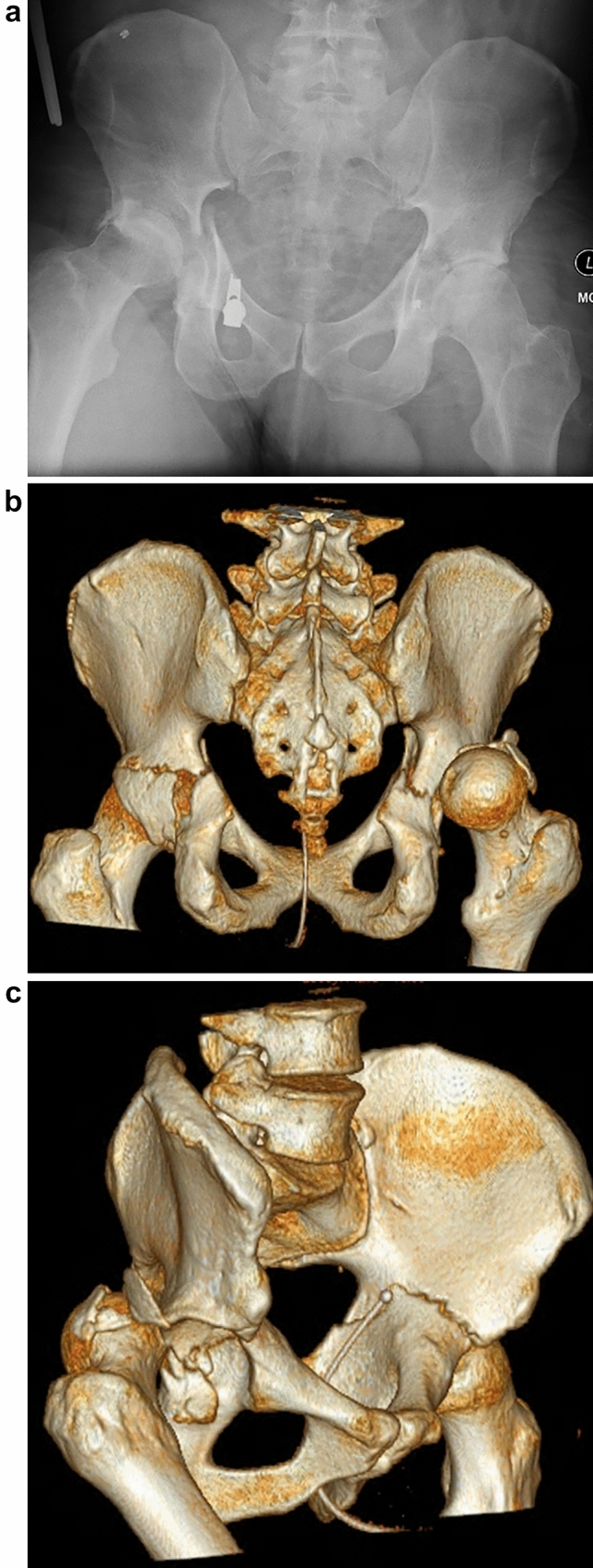
Anteroposterior pelvis radiograph (**a**) and posterior (**b**) and oblique (**c**) 3D surface-rendered CT reconstruction images showing a horizontal shear fracture with associated posterior wall fractures and right-sided posterior hip dislocation

All patients had pelvis radiographs and CT scans. Radiological analysis was performed for all six horizontal shear fracture cases (12 acetabula). In all cases, both transverse acetabular fractures occurred essentially in the same axial plane at a similar level. As viewed on sagittal CT images, the fracture line angle was variable at the anterior column (7 horizontal, 5 oblique) whilst characteristically exiting horizontally at the posterior column (11 horizontal, 1 oblique). Nine of 12 fractured acetabula were displaced (horizontal shear translation ≥ 2 mm, range 2–14 mm). This was measured at the location of maximal displacement. See Table 2 for radiological characteristics. Such displacement was characterised by posterior translation of the antero-inferior half of the pelvic ring (see Fig. 2). In the case of bilateral posterior wall fractures (62B1b), one resulted from posterior hip dislocation (see Fig. 3).

**Table 2:** Radiological characteristics of the horizontal shear fractures

Radiological feature	Number of acetabula
Horizontal shear	
Displaced (≥ 2 mm translation)	9
Undisplaced (0–1 mm translation)	3
Transverse fracture pattern sub-type	
Infratectal (62B1.1)	0
Juxtatectal (62B1.2)	7
Transtectal (62B1.3)	5
Comminution	
Comminuted	10
Not-comminuted	2
Posterior wall	
Fractured	5^a^
Intact	7
Fracture relation to ischial spine	
Above	8
Through	2
Below	2

^a^Bilateral in one patient, unilateral in three patients

### Surgery and post-operative management

**Fig. 4:**
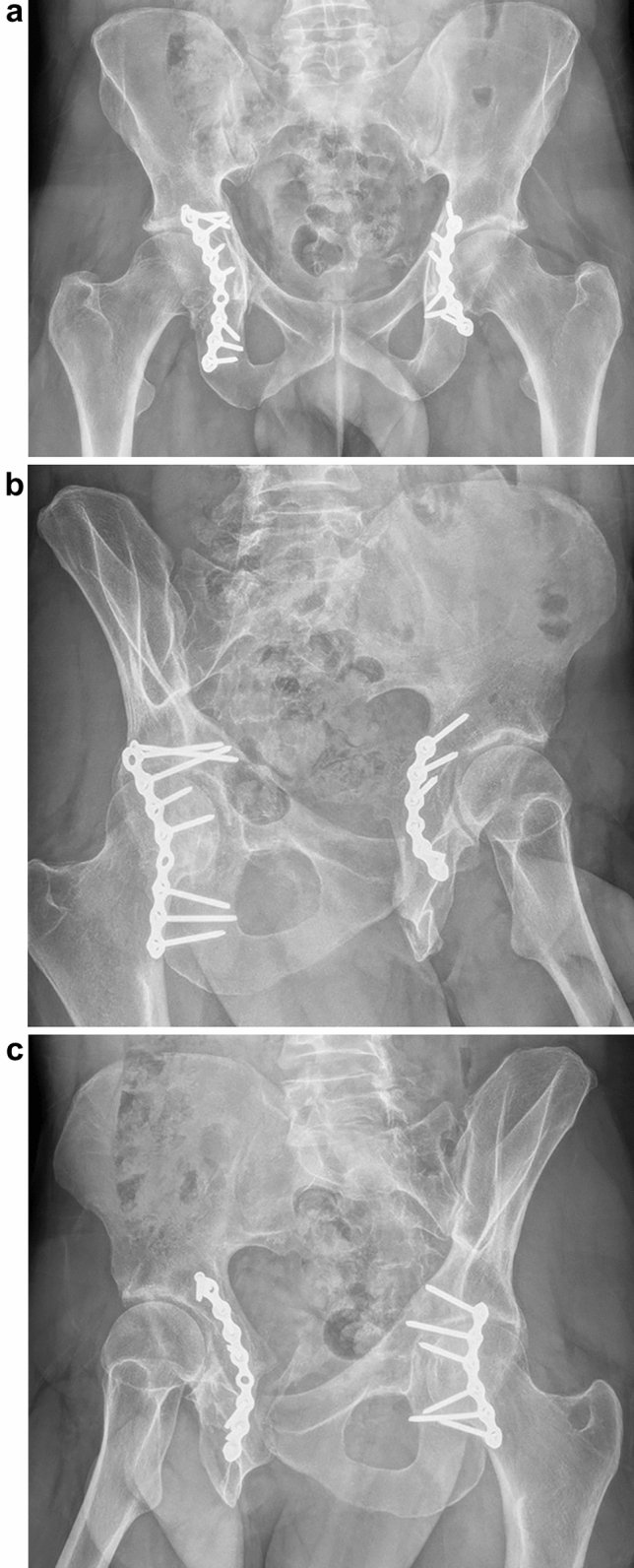
Anteroposterior (**a**) and Judet view radiographs (**b**, **c**) demonstrating osteosynthesis, preservation of hip joint space and united bilateral transverse acetabular fractures at follow-up

An orthopaedic trauma team at a level 1 tertiary centre managed all patients. All nine displaced fractures underwent open reduction and internal fixation (ORIF). Three of the six patients required emergency surgery prior to ORIF. One patient had a temporary pelvic external fixator, definitive femoral intramedullary nailing and thigh fasciotomy, the second had a trauma laparotomy before transfer and then also had an external ventricular drain for intra-cranial haemorrhage on arrival, and the third had a closed reduction of their hip dislocation and skeletal traction. The median time from admission to surgery for ORIF of the horizontal shear fracture was 4 days (range 2–16 days). The patient who did not undergo ORIF was the youngest patient, had an undisplaced horizontal shear fracture, and was managed by 8 weeks of non-weightbearing followed by progressive weightbearing. In operative cases, through a Kocher–Langenbeck approach, the posterior column was fixed using either one or two low-profile pelvic reconstruction plates (De-Puy Synthes, Warsaw, Indiana, USA), see Fig. 4. Posterior wall fractures were reduced and also held with posteriorly placed reconstruction plates. One patient with severely comminuted fractures had ilioinguinal approaches with anterior column fixation using bilateral supra-pectineal plates (Stryker, Kalamazoo, Michigan, USA). No sacral fractures required fixation after the acetabular columns were reduced and stabilised. Post-operatively patients were allowed to sit up in bed, commence bed-based range of motion exercises but were kept non-weightbearing for 6 weeks with privileges for stationary bike riding, swimming, and walking in shoulder depth water. At 6 weeks, repeat radiographs and clinical review occurred at our clinic before commencing walking again with progressively increasing weight. Patients were allowed to return to physical work once fully weightbearing, typically after 3 months. Successive follow-up with serial radiographs occurred through our clinic at 3, 6, 12, and 24 months post-operatively, after 24 months, follow-up was organised only in case of new hip-related symptoms.

#### Outcomes at follow-up

All three patients reporting pain at follow-up had transtectal (62B1.3) type fractures and one had ipsilateral femoral head osteonecrosis, whilst another developed bilateral heterotopic ossification (right; Brooker grade 3 and left; grade 2). This patient suffered severe brain injury, traumatic shock requiring massive transfusion, significantly comminuted fractures, and was the only patient to have bilateral ilioinguinal and Kocher–Langenbeck approaches. Two of the three patients with any degree of post-traumatic arthritis sustained associated posterior wall fractures. Nine of ten hips demonstrated minimal post-traumatic changes according to Matta’s classification. The single hip with a poor grade developed femoral head osteonecrosis managed with total hip arthroplasty at > 1 year post-operatively. Both patients with difficulty mobilising were those who sustained sciatic nerve injuries with foot-drop. One had a tibialis posterior tendon transfer for unilateral foot-drop 1 year after injury and one patient utilised ankle–foot orthoses bilaterally. When comparing fracture and implant position on serial radiographs, no displacement occurred. No patient reported implant complications or underwent a secondary procedure for infection or implant removal. No deaths occurred 1 year after injury. See Table 3 for a summary of clinical outcomes which were recorded at the last clinical review for those five patients with greater than 12 month follow-up (median 22, range 13–94 months).

**Table 3:** Outcomes at follow-up of patients with horizontal shear fracture of the pelvis

Outcome	Number of patients
Pain^a^	
Nil	2
Slight/intermittent pain	1
After walking but resolves	0
Moderate pain but still able to walk	2
Severe, prevents walking	0
Mobilising^a^	
Normal	3
No cane but slight limp	0
Long distance with crutch	0
Limited even with support	2
Very limited	0
Unable to walk	0
Subsequent surgery	
Hip arthroplasty	1 (unilateral)
Other hip or acetabular surgery	0
Other related surgery^b^	1
	*Number of hips*
Radiographic post-traumatic arthritis^a^	
Excellent	5
Good	4
Fair	0
Poor	1

^a^Adapted from Matta Grading System

^b^Tibialis posterior tendon transfer

## Discussion

The horizontal shear fracture of the pelvis is characterised by bilateral transverse acetabulum fractures. This injury is uncommon, accounting for 0.5% of all pelvic and acetabular fractures over 10 years at our institution. In these fractures, the anterior ring typically shears posteriorly ≥ 2 mm relative to the posterior pelvic ring through the bilateral transverse acetabular fractures. This is the first report of this pattern. These fractures are high-energy injuries normally following motorcycle crashes. The proposed mechanism is a large horizontal force transferred through both femora into the acetabula, for example, as the patient hits the ground with flexed hips after coming off the motorcycle or being ejected from a vehicle. Other mechanisms for this injury may include a crush injury with significant weight, for example under a horse, with the hips flexed and a force again directed along both femora into the acetabula. We propose that these specific fractures are rare, because they should require both hips to be concurrently positioned exactly at a similar position of flexion and slight abduction at impact and simultaneously both femora, rather than one, must strike the ground or object together to produce the horizontal shearing injury. The likelihood of this exact combination of events is scarce, and without it, the other more common patterns of unilateral acetabular fractures are instead produced. The horizontal shear fractures are frequently juxtatectal (62B1.2) or transtectal type (61B1.3), comminuted, have horizontal trajectories through the columns, and typically exit above the ischial spine. Posterior wall fractures may also occur. Sacral fractures and sciatic nerve injuries affected half of the patients. The Matta post-traumatic arthritis grade was excellent or good in 90% of hips. Patients who reported any pain or difficulty mobilising at follow-up sustained related sequelae including femoral head osteonecrosis, heterotopic ossification, or foot-drop related to sciatic nerve injury.

This study’s limitations are acknowledged. First, the number of patients with this pattern is small. With larger studies, these initial findings may be expanded upon and the generalisability of findings may be improved. However, it is uncommon, constituting just 0.5% pelvis and acetabular fractures at our institution over 10 years. Matta also found bilateral acetabular fractures accounted for only 1% of patients with acetabulum fractures [5]. Unfortunately, epidemiological studies have not reported the prevalence of bilateral acetabular fractures regardless of country or number of patients, possibly due to their shear rarity [11–16]. This is a retrospective study, and therefore, detailed functional outcomes such as modified Merle d’Aubigné scores are not reported [17]. It would be of interest to compare the functional scores of patients sustaining horizontal shear fractures to other fracture patterns. Follow-up for patients was 1 year; longer follow-up would be valuable to determine if patients remain free of post-traumatic arthritis.

The most prevalent unilateral acetabular fracture type is the posterior wall fracture (20–32%) followed by both column fractures (17–20%) [1, 12, 18]. There is one report of bilateral posterior wall fractures; however, none of bilateral both column fractures [19]. Bilateral acetabulum fractures are most commonly central fracture dislocations [20–23]. These often occur secondary to seizures [20–23], sustained myoclonus [24], or cerebrovascular accident-related convulsions [25]. Central fracture dislocations have occurred with bilateral femoral neck fractures [25]. Seizures also caused bilateral T-type acetabulum fractures in one case [26], whilst osteoporosis caused bilateral anterior column posterior hemi-transverse fractures in another [27]. One study reported on eight cases of bilateral acetabular fractures most commonly following motor vehicle crashes which included two cases of transverse fractures with posterior wall fractures [28]. Bilateral anterior column fractures were reported following motor vehicle crashes [29] and in one paediatric case following an ice-hockey collision [30]. Motor vehicle crashes have also produced an anterior wall fracture with contra-lateral posterior wall fracture [28, 31] and bilateral posterior column and posterior wall fractures [19]. Our aim was to describe this unique pattern, of bilateral horizontal shear lines through the transverse plane of the acetabula separating the pelvic ring into anterior and posterior ring.

Bilateral transverse acetabular fractures are rare with only two case reports to date [6, 7] and this pattern has not been reported in larger epidemiology studies [11–15]. One occurred in a 24-year-old female ejected from her vehicle and another in a 65-year-old pedestrian struck by a car and thrown 20 feet. Such mechanisms were similarly high-energy to this study. As found in our study, associated posterior ring fractures were reported, with the 24-year-old-patient having a left sacral fracture and right sacroiliac joint widening [6]. Traumatic sciatic nerve injuries have a reported incidence of 16% in patients with acetabular fractures [32]. Neither the 24-year-old or 65-year-old sustained sciatic nerve injuries; however, they did not have associated posterior wall fractures and likely did not experience a posterior shear-type mechanism that we found in motorcycle crashes. Pure transverse acetabular fractures are less prevalent (3–9%) than the associated transverse with posterior wall fractures (8–21%) [1, 12, 14, 18]. Transverse with posterior wall fractures occurred in five patients in our group, and unsurprisingly, this pattern is found most commonly with sciatic nerve injury [33]. All fractures were juxtatectal or transtectal, similar to the findings in unilateral transverse acetabular fractures [1].

Two patients in our group required a massive transfusion protocol. One required internal iliac artery angioembolisation. In the previous reports, the 24-year-old patient had blood transfusions but no documented massive transfusion protocol [6]. Of note, no deaths occurred with level 1 trauma centre care in our study and in both case reports of bilateral transverse acetabular fractures [6, 7]. The 24-year-old patient had bilateral sacroiliac joint screw fixation and anterior supra-acetabular external fixator, whilst the 65-year-old’s diagnosis was delayed, having left-sided protrusion managed with skeletal traction [6, 7]. These management options differed to ours. In horizontal shear fractures, the displaced acetabulum needs anatomical reduction and buttressing of the posterior column through internal fixation. This fixation also restores continuity between the anterior and posterior rings.

Published data regarding the follow-up of patients with bilateral transverse acetabular fractures are scarce [6, 7]. We identified one patient with a poor grade of traumatic arthritis with an ipsilateral posterior wall fracture. The prevalence of post-traumatic arthritis following acetabular fracture is 26.6% [32] and posterior wall fractures are reported to have greater risk of post-traumatic arthritis [34]. One case of femoral head osteonecrosis occurred in our group in a hip which was not dislocated. Femoral head avascular necrosis occurs in 5.6% of patients with acetabular fractures and 9% in those with posterior dislocation [32]. The osteonecrosis was managed definitively with total hip arthroplasty. The rate of hip arthroplasty following acetabular fractures that result in post-traumatic arthritis or osteonecrosis is reported at 15% at a median of 4 years [35]. Regarding functional outcomes, a modified Merle d’Aubigné score was not available for our patients. On analysis of 906 patients correlating modified Merle d’Aubigné scores with fracture pattern, in transverse fracture types, 86.3% had excellent or good scores and transverse with posterior wall fractures were excellent or good in 83.0% [32].

Young and Burgess described a mechanism-based classification [3]. The horizontal shear mechanism involves bilateral transverse acetabular fractures with a separation of the anterior and posterior ring. When comparing combined acetabular and pelvic ring injuries to isolated acetabular fractures, transverse fractures and T-type fractures more commonly occur with pelvic ring injuries [36]. This may be explained by transverse type fractures occurring after a high-energy force directed through the acetabulum sufficient to additionally cause posterior ring injuries [37]. Our study supports this, as four of six patients had posterior pelvic ring injuries. Following a motorcycle crash or other significant force directed from anterior to posterior, the horizontal shear pattern can have concomitant posterior wall fractures, hip dislocation, or posterior pelvic ring injury.

In conclusion, we have described the horizontal shear fracture of the pelvis being characterised by transverse fractures through both acetabula causing separation of the anterior and posterior pelvic ring. It is an uncommon fracture and requires significant energy to produce, typically following motorbike crashes. The most common associated injuries include posterior wall fractures, posterior pelvic ring fracture, and sciatic nerve injury. Conceptually, it may fit into a mechanism-based system such as the Young and Burgess classification. Understanding this horizontal shear pattern will facilitate the management of patients with these injuries. Good radiological and clinical outcomes were achieved by the method of treating displaced horizontal shear fractures with open reduction and internal fixation which allowed restoration of the acetabulum, buttressing the posterior column and re-established stable continuity between the anterior and posterior pelvic ring.
